# Effects of the combined use of a probiotic and chromium methionine chelate on the functional state of broiler chickens

**DOI:** 10.14202/vetworld.2023.2358-2365

**Published:** 2023-11-27

**Authors:** Tatiana Kazakova, Olga Marshinskaia

**Affiliations:** Federal Research Center of Biological Systems and Agrotechnologies of the Russian Academy of Sciences, Orenburg, Russia

**Keywords:** antioxidant, broiler chickens, chromium, distilled water

## Abstract

**Background and Aim:**

An increase in the productivity of broiler chickens is possible when creating an optimal food base that provides birds with all of the nutrients and biologically active substances required for the fullest realization of their genetic potential. In this regard, we examined the effects of the addition of a water-based probiotic and a chelated form of chromium (Cr) to the diet of birds.

**Materials and Methods:**

Sixty 14-day-old male Arbor Acres broilers were used in this study. The birds were assigned to two groups of 30 birds according to their body weights. The control broilers received distilled water with the basal diet, and the experimental group received a probiotic preparation in drinking water and Cr methionine chelate (Cr-Met) in the diet. The feeding period lasted 28 days. Growth performance indices were measured throughout the experiment. At the end of the experiment, blood sampling was performed to assess blood biochemical parameters, antioxidant system indicators, and trace elements.

**Results:**

We found that the introduction of a probiotic preparation and a chelated form of Cr into the diet of broiler chickens had a positive effect on meat productivity, which was characterized by a 17% increase in the average daily gain of birds (p = 0.05) and a 14% increase in body weight (p = 0.01). Consequently, the yield of the slaughtered carcass increased by 5.8% (p = 0.05). Against the background of the consumption of the developed diet, broiler chickens exhibited a 14% decrease in feed conversion accompanied by an increase in the level of digestibility of dietary nutrients. In addition, glucose levels were decreased by 20% (p = 0.03) against the background of a 76% increase in the total protein concentration (p = 0.01). Superoxide dismutase and glutathione peroxidase activities were increased by 13% (p = 0.02) and 7.5% (p = 0.03), respectively. Elemental analysis of blood serum revealed a 99% decrease in the Fe level versus the control (p = 0.02) and a 31% increase in the Zn level (p = 0.02).

**Conclusion:**

We conclude that feeding broiler chickens is a multicomponent probiotic supplement combined with Cr-Met promotes growth and nutrient absorption, and optimizes metabolic processes.

## Introduction

The practical experience of scientists has revealed that an increase in the productivity of broiler chickens is possible when creating an optimal food base that provides birds with all nutrients and biologically active substances needed for the complete realization of their genetic potential [1, 2].

Minerals are important in the organization of proper feeding for farm animals [3]. Researchers have obtained new data on the mineral needs of poultry, thereby substantiating the importance of balancing diets for a number of new, previously non-standardized chemical elements, including chromium (Cr) [4–6]. Many studies demonstrated that supplementation with this vital micronutrient in various chemical forms and doses improves the productivity of birds [7, 8]. Prior studies found that the bioavailability of Cr from inorganic compounds in the gastrointestinal tract of animals is low (0.4%–3%); however, its bioavailability increases to 20%–25% when Cr is supplied in the form of complex compounds (picolinates and asparaginates), leading to the increased use of these forms [9, 10]. However, there are no recommendations from the National Research Council regarding the content of Cr in the diets of farm birds [11, 12]. The accumulated theoretical and practical material on the importance of Cr in the feeding of farm animals is the basis for optimizing its content in diets to increase the productive characteristics and maintain the health of birds, an extremely relevant topic worthy of further study.

In addition, scientists in recent decades have paid considerable attention to supplements that affect the intestinal microflora. It is well known that the state of the microflora plays an important role in the immune status, metabolism, and nutrient absorption of animals [13–15]. A number of researchers have reported that probiotics have a multifunctional effect. Their use contributes to the normalization of the bacterial background of the gastrointestinal tract, the suppression of pathogen colonization, and an increase in feed nutrient conversion, and they also improve the growth rates of birds, egg production, and the quality of eggs and eggshells [16, 17].

Previous studies have focused on the combined use of a water-based probiotic but the study on the chelated form of Cr in the diet of birds is not available [18]. This study examined the effects of their combined use on the functional state of broiler chickens.

## Materials and Methods

### Ethical approval

The experimental studies were conducted in accordance with the instructions and recommendations of the Russian regulations (Order of the Ministry of Health of the USSR No. 755 of August 12, 1977 “On measures to further improve the organizational forms of work using experimental animals”), the protocols of the Geneva Convention and the principles of good laboratory practice (National Standard of the Russian Federation GOST R 53434–2009). All procedures on animals were performed in accordance with the rules of the Animal Ethics Committee of the FSSI FRC BST RAS.

### Study period and location

This study was conducted in August 2022. The study was conducted in the laboratory of biological testing and expertise of the Federal State Budgetary Scientific Institution “Federal Scientific Center for Biological Systems and Agrotechnologies of the Russian Academy of Sciences” (accreditation certificate of the State Standard of Russia - PA.RU21ПФ59 dated December 02, 2015). The studies were conducted at the Central Collective Use Center of the FSSI FRC BST RAS (http://цкп-бст.рф.).

### Experimental animals

Sixty 14-day-old male Arbor Acres broilers (CJSC “Orenburgskaya Poultry Farm”) were used in the study. The birds were assigned into two groups of 30 each based on their body weight. Broilers in the control group were given distilled water with the basal diet (BD), and those in the experimental group received a probiotic preparation (Lactobifadolum, Russian Federation) in drinking water with a concentration of 1 × 10^6^ colony-forming units (CFU) of *Lactobacillus acidophilus* and 8.0 × 10^7^ CFU of *Bifidobacterium adolescentis* in 1 g of nutrient medium with supporting component maltodextrin (1% in preparation) and 200 mg/kg Cr methionine chelate (Cr-Met) in the diet. Chromium-met consists of Cr (not <0.1%) chelated with methionine (3.4%), and it supplies the diet with 0.8 mg/kg Cr. The dose of Cr-Met was based on the data of Dalólio *et al*. [19], who found that the optimal dose of Cr in feed to improve performance and carcass characteristics in broilers is 1.08 mg/kg regardless of the source used. This chelated metal dosage was reduced because probiotics increase the digestibility of nutrients in the diet. The feeding period was 28 days.

Birds were fed the same BD according to the nutritional recommendations of the All-Russian Research and Technological Institute of Poultry (Table-1). Feed and drinking water (free of antibiotics) were provided *ad libitum*.

**Table-1:** Ingredients and chemical composition of the basal grower and finisher diets fed to broilers.

Ingredients	Grower feed (15–28 days)	Finisher feed (29–42 days)
Ingredients, %		
Wheat	41.50	71.88
Corn	20.00	-
Soybean meal, 46% CP	20.89	11.45
Soybean oil	6.23	4.55
Meat meal	3.85	3.25
Sunflower oil	4.00	5.50
Salt	0.21	0.25
Monocalcium phosphate	0.56	0.35
Limestone meal	0.46	0.65
Premix*	2.25	1.95
Total	100	100
Calculated composition		
Metabolizable energy, kcal/100g	298	303
Crude protein, %	21.99	19.32
Crude fiber, %	4.06	4.68
Lysine, %	1.36	1.11
Methionine, %	0.50	0.50
Methionine+cysteine, %	0.79	0.76
Calcium, %	0.90	0.86
Total phosphorus, %	0.60	0.60
Sodium, %	0.16	0.16

Note:

* Vitamin and mineral premix provided per kilogram of diet: Vitamin А 12.0 KIU/kg; Vitamin D3 4.0 KIU/kg; Vitamin E 60.0 mg/kg; Vitamin K3 2.0 mg/kg; Vitamin B1 2.0 mg/kg; Vitamin B2 8.0 mg/kg; Vitamin B3 30.0 mg/kg; Vitamin B4 500.0 mg/kg; Vitamin B5 10.0 mg/kg; Vitamin B6 3.0 mg/kg; Vitamin B12 0.025 mg/kg; Vitamin B9 0.5 mg/kg; Vitamin H 0.1 mg/kg, Fe 25.0 mg/kg; Cu 10.0 mg/kg; Zn 70.0 mg/kg; Mn 80.0 mg/kg

The temperature and relative humidity corresponded to the norms recommended for broiler growth. The photoperiod program complied with the European Social Security Regulation 43/2007 (Council Directive 2007/43/EU providing the minimum rules for the protection of chickens kept for meat production).

### Assessment of Cr concentrations in diets

Samples of experimental diets were collected weekly and composited by phase for Cr analysis. Composite feed samples were dried for 48 h at 55°C in a forced air oven and then prepared for Cr analysis by wet ashing with trace metal grade nitric acid. Chromium was determined using a NexION 300D spectrometer (PerkinElmer, Waltham, MA, USA).

### Assessment of growth indicators

The dynamics of growth rates were assessed by individual weighing of birds on days 14, 21, 28, 35, and 42 of the experiment before feeding. Based on the weighing results, the average daily gain was calculated. The feed conversion ratio was calculated on day 42 of the experiment. Mortality was recorded at its occurrence, and the general state of health was monitored during the entire experimental period.

### Assessment of blood parameters

Blood was sampled from the birds from the axillary vein in the morning at the age of 42 days. For blood sampling, vacuum tubes with a blood-clotting activator were used (Greiner Bio-One International AG, Kremsmünster, Austria).

Biochemical analysis was performed on a CS-T240 automatic biochemical analyzer (Dirui Industrial Co., Ltd, Changchun, China) using commercial biochemical kits (Randox, Crumlin, UK). The biochemical analysis included the determination of glucose, total protein, total cholesterol, alanine aminotransferase (ALT), and aspartate aminotransferase (AST).

The state of the antioxidant system was assessed through a colorimetric method by determining the activity of catalase (CAT), superoxide dismutase (SOD), and glutathione peroxidase (GSH-Px) using the following reagents: GSH-Pх Assay Kit (A005), enzyme-linked immunosorbent assay (ELISA) Kit for CAT, and ELISA Kit for SOD. The analysis steps corresponded to the kit instructions.

Elemental analysis of blood serum was performed using a NexION 300D spectrometer (PerkinElmer). Serum samples were diluted with acidified diluent consisting of 1% 1-butanol (Merck KGaA, Darmstadt, Germany), 0.1% Triton X-100 (Sigma-Aldrich, Co., St. Louis, MO, USA), and 0.07% HNO_3_ (Sigma-Aldrich) in distilled deionized water (18 MOM cm^−1^; Merck Millipore, Billerica, MA, USA). The content of vital microelements (Co, Cr, Cu, Fe, I, Li, Mn, Se, Si, V, Zn) was determined in the samples.

### Assessment of carcass characteristics

At the end of the experiment, the birds were weighed to the nearest gram and slaughtered. The carcass without giblets was weighed, expressed as a percentage of its live body weight, and the carcass yield was considered. In addition, the weights of the liver (without gallbladder) and heart were determined.

### Statistical analysis

All data were analyzed using Statistica version 10 (StatSoft Inc., Tulsa, OK, USA). The normality of the obtained data was checked using the Shapiro-Wilk test. The hypothesis that the data were normally distributed was rejected in all cases with a probability of 95%, which justified the use of non-parametric procedures for downstream statistical analyses. Therefore, we examined differences between the groups using the Mann–Whitney U test. The obtained data are presented as the median and 25^th^–75^th^ percentiles. For all statistical analyses, the achieved significance level was calculated, and the critical level of significance in this study was p ≤ 0.05.

## Results

In the analysis, the average Cr level in the control diet was 0.39 mg/kg, compared to 1.23 mg/kg in the experimental diet (Table-2).

**Table-2:** Analyzed chromium concentrations in diets, mg/kg.

Groups	Supplemental Cr, mg/kg	Analyzed Cr	

		Grower feed (15–28 days)	Finisher feed (29–42 days)
Control	0	0.33	0.45
Experiment	0.8	1.14	1.32

Note: *(p ≤ 0.05); ** (p ≤ 0.01) – p-level comparing experimental group I with control group

The dynamics of their growth were monitored during the entire period of chicken growth (Table-3). Broiler chickens in the experimental group were characterized by sufficiently high growth energy, and by the time the breeding was completed (42 days old), the weight of the birds significantly exceeded the control value by 14% (p = 0.01). In the birds of the experimental group, an intensive increase in body weight was observed, as indicated by a significantly higher average daily gain than observed for control birds (17%, p = 0.05).

**Table-3:** Zootechnical indicators of broiler chickens.

Age of broilers, days	Groups	

	Control	Experiment
Body weight, g		
14 days	455.0 (440.0–475.0)	467.0 (442.0–480.0)
21 days	800.0 (766.0–834.0)	900.0 (860.0–910.0)
28 days	1228.0 (1010.0–1356.0)	1446.0 (1 2221.0–1532.0)*
35 days	1667.0 (1668.0–1800.0)	1999.0 (1952.0–2222.0)
42 days	2266.0 (2207.0–2345.0)	2596.0 (2451.0–2679.0)**
Weight gain, g/days		
14–42 days	64.8 (62.2–66.7)	76.0 (71.7–78.5)*

Note:

* (p ≤ 0.05);

** (p ≤ 0.01) – p-level comparing experimental group I with control group

Feed constitutes a significant part of the cost structure of poultry products. Accordingly, daily accounting of feed consumption was performed during the entire growing period (Table-4). Supplementation with probiotics and Cr-Met resulted in increased feed intake on days 28 and 42.

**Table-4:** Feed consumption of broiler chickens.

Age of broilers, days	Groups	

	Control	Experiment
Feed intake, g/bird		
14–28	1634.0	1723.0
28–42	2530.0	2455.0
Total	4164.0	4178.0
Feed conversion ratio, kg		
14–42	2.2	1.9

Based on the findings, it can be concluded that the use of this supplementation contributed to a decrease in feed costs per 1 kg of bird growth. For example, in the experimental group, this indicator was 0.3 kg lower (14%) than that in the control group.

The recorded differences in the live weight of broiler chickens can be explained by the digestibility of dietary nutrients. It should be noted that the birds in both groups exhibited a fairly high level of digestibility of dietary nutrients (Table-5).

**Table-5:** Digestibility coefficients of feed ingredients, %.

Digestibility coefficient	Groups	

	Control	Experiment
Organic matter	76.0 (75.1–76.5)	80.0 (78.1–80.4)
Crude protein	78.2 (77.4–78.6)	80.9 (79.1–81.2)*
Crude fat	73.1 (72.9–74.2)	74.5 (74.0–75.6)
Crude fiber	13.3 (12.3–14.9)	13.8 (12.1–14.0)
Dry matter	76.9 (75.8–77.1)	78.2 (77.3–78.4)
NFE	78.2 (76.3–80.1)	82.0 (81.1–83.2)

NFE=Nitrogen-free extractive

In the control group, the digestibility of dry matter was 76.9%, whereas it was 1.3% higher in the experimental group. A similar pattern was observed regarding the digestibility of organic matter. The digestibility of crude protein was 2.7% higher in the experimental group (p = 0.03). The digestibility of nitrogen-free extractive substances in broiler chickens was 3.8% higher in the experimental group than in the control group. The digestibility of crude fat and fiber did not practically differ between the groups.

This analysis indicated that the higher live weight of the broilers in the experimental group predetermined the higher meat productivity of birds (Table-6).

**Table-6:** Carcass characteristics of broilers.

Item	Groups	

	Control	Experiment
Live body weight, g	2266.0 (2207.0–2345.0)	2596.0 (2451.0–2679.0)**
Semi-eviscerated carcass, g	1984.0 (1903.3–2018.3)	2275.0 (2110.0–2360.1)
Eviscerated carcass, g	1539.0 (1447.0–1588.1)	1914.4 (1884.0–1970.8)*
Liver, g	75.09 (74.64–84.46)	82.96 (79.07–87.29)
Heart, g	14.76 (13.59–14.77)	15.77 (14.05–16.04)
Carcass yield, %	67.9 (65.6–68.1)	73.7 (72.9–74.5)*

Note:

* (p ≤ 0.05);

** (p ≤ 0.01) – p-level comparing experimental group I with control group

The results for carcasses illustrated that the weight of the gutted carcass was 24% higher in the experimental group than in the control group (p = 0.05). In addition, the slaughter yield was significantly higher in the experimental group (5.8%, p = 0.05). There were no statistically significant differences between the groups in terms of the offal mass (liver and heart).

The chemical composition was determined for a comparative assessment of the effect of the developed supplementation on the quality and nutritional value of meat using the superficial pectoral muscle as an example (Table-7). The protein content was 2.76% higher in the experimental group than in the control group (p = 0.03). The fat level in the meat in the control and experimental groups was within the recommended ranges [20].

**Table-7:** Content of chemicals in broiler meat, %.

Item	Group	

	Control	Experiment
Moisture	75.26 (75.18–75.3)	75.62 (75.41–76.26)
Dry matter	24.6 (24.47–24.74)	25.15 (24.9–25.55)
Protein	19.57 (19.17–20.75)	22.33 (20.17–22.57)*
Fat	5.4 (4.95–5.62)	5.02 (5.01–5.11)
Ash	0.95 (0.94–0.96)	0.95 (0.94–0.96)

Note:

* (p ≤ 0.05); ** (p ≤ 0.01) – p-level comparing experimental group I with control group

Biochemical analysis revealed that the blood parameters of all birds were within the recommended limits [21] (Table-8). As presented in the table, the glucose content in broilers in the experimental group was 20% lower than that in the control broilers (p = 0.03), whereas the total protein level significantly exceeded the control level by 76% (p = 0.01). Cholesterol levels tended to be lower in the experimental group. It should be noted that the activities of ALT and AST in the birds in the experimental group significantly exceeded the control values by 8.5% (p = 0.02) and 15% (p = 0.02), respectively, while remaining within the reference values (Table-8).

**Table-8:** Blood parameters of broilers.

Item	Groups	

	Control	Control
GLU, mmol/L	16.19 (15.1–17.24)	13.44 (11.89–14.06)*
Total protein, g/L	41.13 (40.37–42.27)	72.25 (71.93–72.91)**
Cholesterol, mmol/L	1.45 (1.17–1.62)	1.05 (0.86–2.12)
ALT, U/L	19.03 (18.60–20.58)	20.65 (18.1–22.8)*
АST, U/L	152.66 (120.69–203.84)	175.86 (165.37–186.23)*
SOD, U/mL	12.62 (11.93–13.21)	14.29 (14.03–14.53)*
CAT, U/mL	4.56 (4.31–4.78)	4.99 (4.57–5.28)
GSH-Px, U/mL	209.87 (205.36–212.37)	225.64 (220.34–228.42)

GLU=Glucose, CAT=Catalase, ALT=Alanine aminotransferase, АST=Aspartate aminotransferase, SOD=Superoxide dismutase, GSH-Px=Glutathione peroxidase Note:

* (p ≤ 0.05);

** (p ≤ 0.01) – p-level comparing experimental group I with control group

During the experiment, a stimulating effect on the activity of antioxidant enzymes was established. It was found that the activities of SOD and GSH-Px in broiler chickens in the experimental group exceeded the control levels by 13% (p = 0.02) and 7.5% (p = 0.03), respectively, and CAT activity tended to be higher in the experimental group.

**Figure-1:**
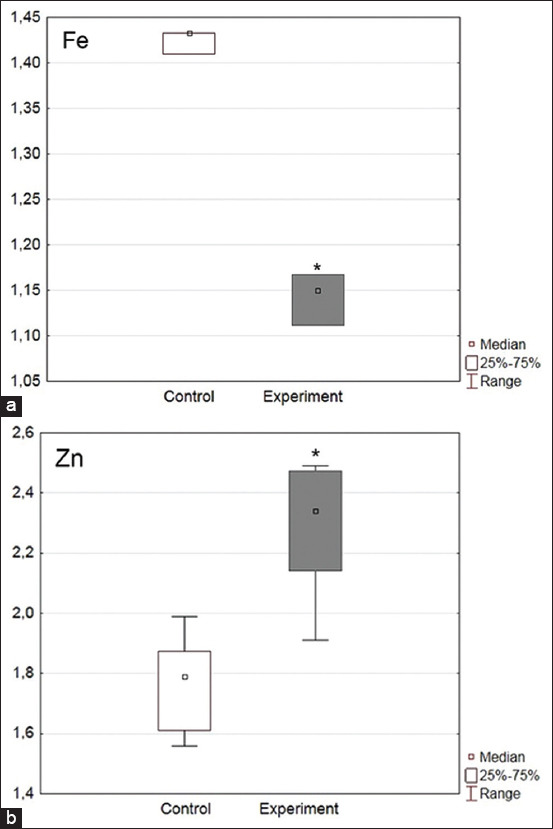
(a and b) Level of Fe and Zn in broiler chicken serum, μg/mL.

**Figure-2:**
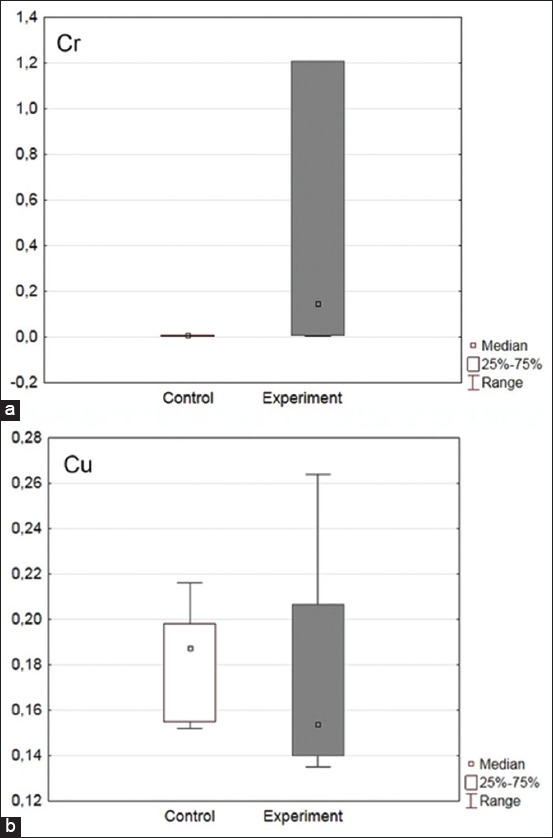
(a and b) Level of Cr and Cu in broiler chicken serum, μg/mL.

The elemental analysis of blood serum revealed a 99% decrease in the Fe level in the experimental group relative to the control level (p = 0.02), whereas the Zn content was increased by 31% (p = 0.02, Figures-1a and b). It should be noted that the Cr and Cu levels were noticeably higher in the experimental group, but the obtained data were unreliable (Figures-2a and b).

## Discussion

The combined use of a probiotic and a chelated form of Cr positively affected broiler chickens’ meat productivity, which was characterized by increases in the average daily gain of birds and body weight, resulting in an increased slaughtered carcass yield. The obtained results are consistent with a number of global studies in which the inclusion of various forms of Cr in feed contributed to an increase in body weight gain in animals and improvements in carcass characteristics and meat quality [22–25]. In particular, Arif *et al*. [26] found that supplementation with 400 ppb Cr propionate in the diet of broiler chickens had a beneficial effect on bird growth and the feed conversion ratio. The increase in body weight is justified by the high activity of metabolic processes in the bodies of broiler chickens occurring against the background of the consumption of the developed additive. Chromium is an essential trace element and a permanent component of the cells of all organs and tissues that perform a number of important functions in the body, including the regulation of carbohydrate, fats, and protein metabolism [27]. Chromium is an active component of glucose tolerance factor, which can increase the metabolic activity of insulin and thereby stimulate the formation of glycogen and the synthesis of fats and proteins, which are used for energy and building functions [28, 29]. In addition, a number of scientists have found that the use of probiotic supplements also affects the change in the dynamics of the live weight of birds and the intensity of their growth to a certain extent [30, 31]. In the course of the study, it was found that the feed conversion ratio was lower in the broilers of the experimental group, which is justified by an improvement in the digestibility of nutrients and the efficiency of their use. This was confirmed by the analysis of nutrient digestibility coefficients. The digestibility of organic matter, protein, fat, and fiber was higher in the experimental group. It should be noted that the effects of Cr supplementation on feed conversion have been inconsistent in the literature. Similar results have been observed in the previous study [32]; however, a previous study did not reveal a significant effect of Cr supplementation on feed conversion [33]. The positive effect observed in our study is associated with the introduction of a multicomponent probiotic into the diet of birds. A number of scientists have revealed that the digestibility and availability of nutrients largely depend on the obligate microbiota [34, 35]. It has been established that introducing probiotics into the diet of birds contributes to the restoration of intestinal microflora, improves the functioning of the digestive system via the additional production of enzymes in the digestive tract, and enhances the absorption of nutrients [36, 37]. In addition, Cr supplements can increase the sensitivity of tissue receptors to insulin, which leads to an increase in glucose uptake by cells and an improvement in appetite [38].

By analyzing the data of the biochemical blood test, we can conclude that complete carbohydrate and protein metabolism was more complete in the experimental group than in those in the control group. The lower serum glucose level in birds that received probiotic supplements in combination with Cr-Met in our study might have indicated an increase in the metabolism and utilization of glucose in cells and tissues because of increased insulin activity induced by Cr [39]. In research by Patil *et al.*, [40], it was revealed that the glucose concentration in blood serum was significantly reduced in broilers treated with Cr picolinate (CrPic) at dosages of 200, 400, and 600 ppb compared to the findings in the control group. Aslanian *et al*. [41] studied the effects of Cr-Met supplementation and observed a decrease in the glucose concentration. It should be noted that the metabolic effects of insulin are diverse, and this hormone affects both carbohydrate and protein metabolism, thereby accelerating anabolic processes. Insulin activates protein synthesis in the liver, muscles, and heart and reduces their breakdown. This fact was confirmed by a significant increase in the protein level in the blood and superficial pectoral muscles of the birds in the experimental group. In addition, these results might be associated with increased nutrient absorption. This study revealed that Cr-Met supplementation at a dose of 200 mg/kg led to significant increases in the activities of ALT and AST in the blood serum of birds in the experimental group, although these indicators remained within their recommended ranges [20]. In this regard, increases in the activities of ALT and AST in blood serum on the addition of Cr-Met should not be considered a side effect.

This study found that the addition of a probiotic supplement in combination with Cr-Met to the diet of broiler chickens had a stimulating effect on the activities of SOD and GSH-Px. An increase in the activity of antioxidant enzymes under the action of Cr occurs because of both the synthesis of new molecules of antioxidant enzymes and the direct activation of their enzymatic activity [42]. In experimental studies, scientists noted that lipid peroxidation processes are enhanced with insufficient dietary Cr intake, and the enzymatic link of antioxidant protection is weakened [43]. By contrast, the addition of Cr complexes to the diet reduces oxidative stress and increases the activity of the antioxidant system [44, 45]. These facts indicate the indispensability of Cr in nutrition and emphasize its special biological role in enhancing the antioxidant defense of the body.

Elemental analysis of blood serum did not reveal significant differences in the level of Cr in the blood serum of birds between the control and experimental groups, although it tended to be higher in the experimental group. The decreases in the concentrations of Fe and Cu were possibly attributable to the fact that Cr competes with these trace elements for the binding sites of transport proteins (transferrin). Because of this competition, the levels of these minerals in blood are decreased [46]. It has been reported that Cr, by affecting the metabolism of Fe, can have a negative effect on the process of erythropoiesis and contribute to the development of anemia [47]. The scientific literature describes a case of hemolytic anemia and thrombocytopenia caused by long-term use of high doses of CrPic [48].

## Conclusion

The study results indicate that feeding broiler chickens a multicomponent probiotic supplement combined with Cr-Met helps increase growth, assimilate nutrients, and optimize metabolic processes. It should be noted that a decrease in Fe content was recorded in birds that consumed the supplement. Therefore, to improve the formula of the feed additive, it is proposed further to include a chelate complex of amino acids with iron.

## Data Availability

The authors can confirm that all relevant data are included in this article.

## Authors’ Contributions

OM and TK: Conceptualized and designed the study. OM: Material preparation, data collection, and analysis. TK: The first draft of the manuscript. OM: revised the manuscript. Both authors have read, reviewed, and approved the final manuscript.
